# Examining models of telemedicine use among U.S. physicians during the COVID-19 pandemic

**DOI:** 10.1371/journal.pone.0331832

**Published:** 2025-09-08

**Authors:** Bridget Xia, Bradford S. Pierce, Paul B. Perrin

**Affiliations:** 1 School of Data Science, University of Virginia, Charlottesville, Virginia, United States of America; 2 James A. Haley Veterans Affairs Medical Center, Tampa, Florida, United States of America; 3 Department of Psychology, University of Virginia, Charlottesville, Virginia, United States of America; Shiraz University of Medical Sciences, IRAN, ISLAMIC REPUBLIC OF

## Abstract

The purpose of this study is to apply the theory of reasoned action (TRA) and technology acceptance model (TAM) to U.S. physicians’ adoption of telemedicine during the COVID-19 pandemic. A total of 230 physicians licensed in the U.S. completed a cross-sectional survey during the COVID-19 pandemic assessing telemedicine use and components of the TRA and TAM. Path models representing the TRA, TAM, and a trimmed version of the TAM were tested. The TRA was an adequate to poor fit for modeling physicians’ decisions concerning telemedicine use. The TAM demonstrated better fit, although a trimmed TAM was ultimately retained for parsimony with mostly good or adequate fit indices. Subjective norms, perceived usefulness, and attitude toward telemedicine may be important targets for trainings and advocacy efforts to facilitate and sustain telemedicine use in a post-pandemic context.

## Introduction

The COVID-19 pandemic transformed the telehealth landscape in 2020, resulting in accelerated adoption and implementation of telehealth services [1]. In 2019, less than 1% of outpatient visits were conducted via telehealth in the U.S. [2]. Before the pandemic, telehealth was a less appealing option for physicians, and reasons contributing to telemedicine adoption hesitancy and challenges included varied insurance coverage and lower reimbursement rates, strict regulations, technical and logistic barriers, and concerns about quality of virtual care [3]. The public health emergency was declared on January 31, 2020, as a response to the pandemic, allowing the U.S. government to rapidly expand the accessibility and coverage of telehealth [4]. Despite clinical, legal, and technological challenges in telehealth adoption [5], telemedicine was widely accepted and implemented, as it significantly improved access to care and promoted continuity of care in chronic disease management during the pandemic [6].

The rapid uptake of telemedicine services across medical specialties was multifactorial. While removing systemic barriers to practicing telemedicine facilitated its successful implementation, changes in physician behavior also played an important role in the context of decision making and technology adoption. The theory of reasoned action (TRA) and technology acceptance model (TAM) were previously used to examine psychologists’ telehealth use before the pandemic [7] and during the pandemic [8]. These theoretical models of behavior change may also explain the accelerated adoption of telemedicine among physicians during the pandemic.

The theory of reasoned action (TRA) is a social psychology model used to account for the relationship between behaviors and attitudes and has been extensively used to understand and explain health behavior [9]. The TRA suggests that behavioral intentions determine and predict behavior change and that one’s intention to perform a behavior is influenced by both their attitude toward engaging in the behavior and subjective/social norms. [9]. In other words, attitudes and social norms can strengthen behavioral intentions, thereby facilitating behavior change, and more positive behavior-specific attitudes and social norms are likely to lead to greater behavioral intentions [9]. According to the TRA, physicians’ adoption of telemedicine during the pandemic might have been due to their positive attitude toward telemedicine and the subjective norms that approve of telemedicine use.

An alternative behavior change model is the technology acceptance model (TAM) which was adapted from the TRA and has been used to understand the reasons why technology users adopt a new technology [10]. The TAM assesses variables related to technology adoption including perceived ease of use, perceived usefulness, and behavioral intention to use [10]. While both theoretical frameworks suggest that one’s attitude toward technology affects their behavioral intention to use it, the TAM specifically focuses on understanding factors that promote or hinder technology use to explain and predict technology adoption by users [10–13]. The TAM purports that external variables (system design elements and usability features) influence beliefs (perceived ease of use and perceived usefulness), which in turn shape users’ attitudes toward technology use, affecting technology use behavior [10,14]. According to the TAM, a person’s specific attitude toward technology use matter more than subjective/social norms, and as a result, positive attitude toward technology use would have a bigger impact on promoting technology adoption behavior than subjective norms. In the context of telemedicine adoption, physicians’ technology adoption during the pandemic might have been driven by their belief that telemedicine would benefit patient care and improve clinical efficiency and that telehealth technologies were easy to use.

Prior studies have used the TRA, TAM, and a combined model to examine the adoption of telehealth among psychologists during the COVID-19 pandemic [7,8], whereas these models have not been applied to physicians’ telehealth adoption. Examining physicians’ telemedicine uptake during the pandemic through behavior change models is crucial for understanding factors that affect the adoption and sustained use of telehealth technologies. As a result, the purpose of the current study was to assess the applicability of the TRA and TAM to U.S. physicians’ adoption of telemedicine during the COVID-19 pandemic.

## Materials and methods

### Participants

Cross-sectional data collection took place from May 12, 2020 to July 25, 2020 as part of a larger study. Physician participants were recruited across the U.S. via email invitations to participate in this survey study using publicly available information on hospital and clinic websites and directories of professional organizations, social media groups, and professional newsgroups. To be eligible for the study, the participant must have been a licensed, practicing physician in the U.S. and aged 18 years or older. Email invitations were sent to 850 potential participants whose emails were pulled from clinic websites by the study team with invitations also posted in online physician groups. Forty-six invitations were undeliverable. A total of 315 individuals opened the survey, while 21 left after reviewing the information sheet or declining informed consent. The final sample consisted of 228 licensed, practicing physicians after excluding the ones who were ineligible or had dropped out. The survey response rate was deemed acceptable based on prior research on survey research response rates [15]. Physician participants had an average age of 46.21 years, and had been in practice for 18.27 years on average. The majority of the sample identified as women (64%) and White (75%) and practiced in urban and suburban areas (92.5%). Academic medical center was the mostly commonly selected practice setting (39.5%). Sample characteristics appear in Table 1.

**Table 1:** Sample Characteristics (N = 228).

Characteristics	(N = 228)
Age M (SD)	46.14 (10.12)
Years in Practice M (SD)	18.32 (10.00)
Gender N, (%)	
Man	82 (36%)
Woman	146 (64%)
Race/Ethnicity N, %	
White/European-American	170 (75%)
Black/African-American	6 (2.6%)
Hispanic/Latino	9 (3.9%)
Asian/Asian-American	31 (13.6%)
Multiracial/Multiethnic	8 (3.5%)
Other	3 (1.3%)
American Indian/AlaskaNative/Native American	1 (0.4%)
Geographic Setting of Practice N, %	
Urban	152 (66.7%)
Suburban	59 (25.9%)
Rural	17 (7.5%)
Primary Practice Setting N, %	
Hospital	58 (25.4%)
Veterans Affairs Medical Center	7 (3.1%)
Academic Medical Center	90 (39.5%)
Trauma Center	0 (0%)
Health MaintenanceOrganization	1 (0.4%)
Correctional Facility	0 (0%)
Geriatric Facility	0 (0%)
Individual Practice	6 (2.6%)
Group Practice	33 (14.5%)
Outpatient Treatment Facility	7 (3.1%)
Rehabilitation Center	0 (0%)
Residential Treatment Facility	0 (0%)
School/University	8 (3.5%)
Other	18 (7.9%)
Number of Providers in Practice N, %	
One	8 (3.5%)
Two to Five	47 (20.6%)
Six to Ten	52 (22.8%)
Eleven to Twenty	29 (12.7%)
Twenty-one to Fifty	21 (9.2)
More than Fifty	70 (30.7)
Not Reported	1 (0.4%)

### Ethics statement

The Virginia Commonwealth University Institutional Review Board (IRB) approved the study procedures under protocol HM20019315 and deemed the study IRB-exempt. Thus, the need for consent was waived, and no consent documentation was required. An information sheet was available for participants to review.

### Procedures

The initial recruitment email invited physicians to participate in a survey “to help understand potential changes to healthcare treatment approaches as a result of the COVID-19 pandemic” without specifying that the study was about telemedicine use. A reminder recruitment email was sent to physicians who had not completed the survey one week after the initial invitation. By clicking on the link in the email invitation, potential participants were taken to the information form on Qualtrics. After they agreed to participate, participants answered questions about their demographic and professional characteristics, read the definition of telemedicine, and then answered questions about telemedicine use. In the current study, telemedicine was defined as “the use of real-time audio (e.g., telephone) and/or video conferencing technology to provide healthcare services.”

### Measures

#### Demographic and professional characteristics.

Participants provided information about their demographic (e.g., age, gender, race/ethnicity) and professional characteristics (e.g., years of practice, practice setting, medical specialty).

#### TRA and TAM.

Chau and Hu [16] developed an 18-item pool to assess healthcare professionals’ acceptance of technology in healthcare provision using variables such as attitude, subjective norms, perceived behavioral control, perceived usefulness, perceived ease of use, and behavioral intention. To mitigate attrition, six items with the highest factor loading value for each construct were selected from the original item pool and adapted for this study. The item assessing attitude read “Using telemedicine in patient care and management is a good idea.” The item assessing subject norms read “People who are important in assessing my patient care and management think that I should use telemedicine.” The item assessing perceived behavioral control read “If I wanted to, I would have the ability to use telemedicine in my patient care and management.” The item assessing perceived usefulness read “Using telemedicine can improve my patient care and management.” The item assessing perceived ease of use read “I find telemedicine easy to use.” The item assessing behavioral intention read “To the extent possible, I would use telemedicine in my patient care frequently.” Items were measured using a 7-point Likert-type scale from 1 (strongly disagree) to 4 (neutral) to 7 (strongly agree), and the term “telemedicine” replaced “telemedicine technology” in the items. Telemedicine use was measured by the question “What percentage of your patient treatment is provided using telemedicine?” with potential responses from 0–100% in increments of 10%.

While Chau and Hu [16] used their items to examine the TAM and Theory of Planned Behavior (TPB), they noted that the perceived behavioral control scale which was derived from the TPB had weak reliability (Cronbach’s α was.55). As a result, a decision was made to limit model testing to the TRA and TAM only while using the full set of items from Chau and Hu [16], since they had already established the reliability and validity of the constructs of interest to examine technology adoption among healthcare professionals.

### Data analysis

Using IBM AMOS 26.0 [17], path models were developed and analyzed to test associations among variables representing attitudes toward telemedicine, subjective norms, intention to use telemedicine, perceived ease of use, perceived usefulness, and use of telemedicine among licensed physicians practicing in the U.S. Two models were tested initially to correspond with the TRA and TAM. Commonly used fit indices [18] were used to test each model. These include well-known indices such as the chi-squared divided by the degrees of freedom (CMIN/DF), goodness of fit index (GFI), adjusted goodness of fit index (AGFI), normed fit index (NFI), incremental fit index (IFI), and Tucker-Lewis index (TLI). Based on previous literature [19,20], a cutoff of at least.90 for adequate fit was used for the GFI, AGFI, NFI, IFI, and TLI. Akaike information criterion (AIC) and Bayesian information criterion (BIC) were used to evaluate difference of fit among models, with better fitting models exhibiting lower values [18]. Other common indices include root mean squared error of approximation (RMSEA) of.1 or less [21], chi-square to degrees of freedom ratio of less than 2.0, and a comparative fit index (CFI) of more than.90 [20].

## Results

### Model 1: TRA

**Fig 1:**
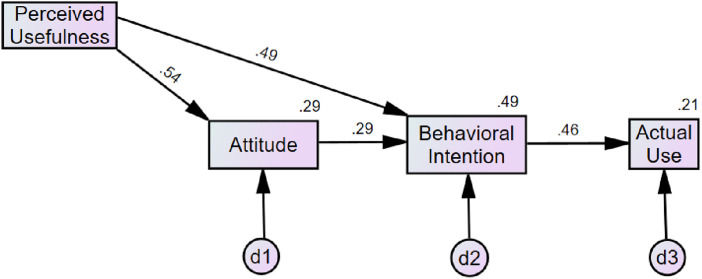
Theory of Reasoned Action (TRA). *Note*. d1 represents the disturbance (error) term for Behavioral Intention, accounting for the variance not explained by Attitude and Subjective Norms; d2 represents the disturbance term for Actual Use, accounting for the variance not explained by Behavioral Intention. The numbers at the top right corner of each box represent the percent variance explained by the prior predictors in the model. All path coefficients are standardized betas.

The TRA (Fig 1) explained 47.1% of the variance in behavioral intention and 21.2% in current use of telemedicine. Within this model, attitude toward using (β = .39, *p* < .001) and subjective norms concerning telemedicine (β = .43, *p* < .001) were uniquely associated with behavioral intention to use telemedicine. Behavioral intention to use telemedicine (β = .46, *p* < .001) was uniquely associated with the current use of telemedicine. There were indirect (mediational) effects of behavioral intention to use telemedicine on the relationships between attitude towards using and current use of telemedicine (β = .18, *p* < .001), as well as between subjective norms and current use of telemedicine (β = .20, *p* < .001). Most fit indices (Table 2) indicated this model was an adequate to good fit for explaining telemedicine adoption of during the COVID-19 pandemic, though RMSEA indicated the TRA was less than a good fit [19,20,22].

**Table 2:** Model fit indices of TRA, TAM, and trimmed TAM.

Fit Index	TRA	TAM	TAM Trimmed
CMIN/DF	11.26	7.10	5.66
GFI	.98	.99	.99
AGFI	.88	.96	.94
NFI	.96	.98	.98
RFI	.87	.95	.94
IFI	.96	.99	.99
TLI	.89	.98	.96
CFI	.96	.99	.99
RMSEA	.14	.06	.09
AIC	27.26	29.10	21.66
BIC	54.76	66.92	49.16

*Note*. Abbreviations: AGFI, adjusted goodness of fit index; AIC, Akaike Information Criterion; BIC, Bayesian Information Criterion; CFI, comparative fit index; CMIN/DF, chi-squared divided by the degrees of freedom; GFI, goodness of fit index; IFI, incremental fit index; NFI, normed fit index; RFI, relative fit index; RMSEA, root mean square error of approximation; TLI, Tucker–Lewis Index.

### Model 2: TAM

**Fig 2:**
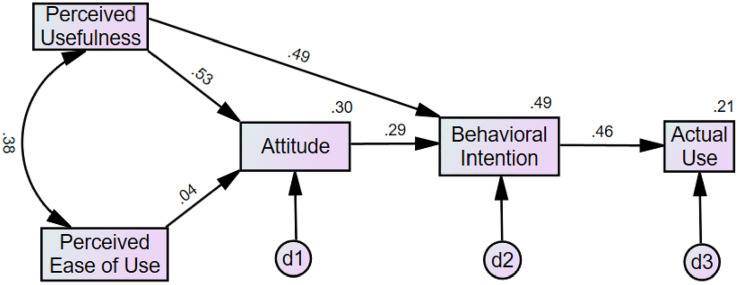
Technology Acceptance Model (TAM). *Note*. d1 represents the disturbance (error) term for Attitude, representing variance not explained by Perceived Usefulness and Perceived Ease of Use; d2 represents the disturbance term for Behavioral Intention, representing variance not explained by Attitude and Perceived Usefulness; d3 represents the disturbance term for Actual Use, representing variance not explained by Behavioral Intention. The numbers at the top right corner of each box represent the percent variance explained by the prior predictors in the model. All path coefficients are standardized betas.

The TAM (Fig 2) explained 29.6% of the variance in attitudes toward telemedicine, 48.6% in behavioral intention, and 21.2% in current use of telemedicine. Within this model, perceived usefulness of telemedicine was associated with attitude toward using (β = .53, *p* < .001), although perceived ease of use was not (β = .04, *p* = .460). Perceived usefulness of telemedicine was positively associated with behavioral intention to use telemedicine (β = .49, *p* < .001), as was attitude towards telemedicine (β = .29, *p* < .001). Finally, as before in the TRA (given the same path being specified), behavioral intention to use telemedicine was associated with current use of telemedicine (β = .46, *p* < .001). Fit indices for this model suggested the TAM was a good fit (Table 2) for understanding telemedicine adoption [19,20,22], generally with better fit indices than the TRA, although some degree of caution should be exerted when comparing fit indices of path models with slightly different variables included.

### Model 3: TAM trimmed

For model 3, the non-significant path (the path between perceived ease of use and attitude towards telemedicine) from the TAM was trimmed (removed) to improve model parsimony, following steps for trimming paths [21]. This simplified version of the original TAM was developed to customize the model to fit the study context by removing statistically insignificant paths. The resulting model contained fewer variables and focused only on the key predictor (perceived usefulness) to directly predict behavioral intention and actual use.

**Fig 3:**
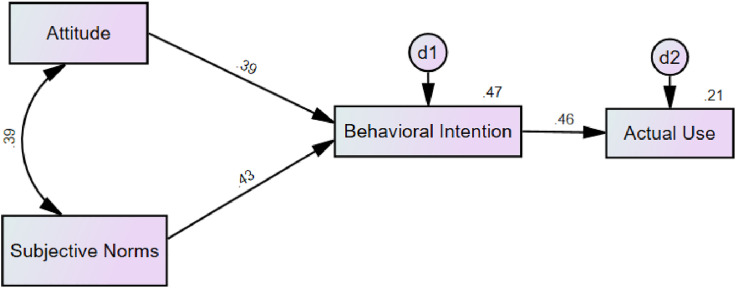
TAM Trimmed. *Note*. d1 represents the disturbance (error) term for Attitude, representing variance not explained by Perceived Usefulness; d2 represents the disturbance term for Behavioral Intention, representing variance not explained by Attitude and Perceived Usefulness; d3 represents the disturbance term for Actual Use, representing variance not explained by Behavioral Intention. The numbers at the top right corner of each box represent the percent variance explained by the prior predictors in the model. All path coefficients are standardized betas.

The trimmed TAM (Fig 3) also explained 29.6% of the variance in attitudes toward telemedicine, 48.6% in behavioral intention, and 21.2% in current use of telemedicine. Within this trimmed model, perceived usefulness of telemedicine was uniquely associated with attitudes toward using (β = .54, *p* < .001) and behavioral intention to use telemedicine (β = .49, *p* < .001). Attitude toward telemedicine was positively associated with behavioral intention to use telemedicine (β = .29, *p* < .001), which positively associated with current use of telemedicine (β = .46, *p* < .001). Attitude toward using telemedicine also had a significant indirect effect on the relationship between perceived usefulness and behavioral intention to use telemedicine (β = .16, *p* < .001). Behavioral intention had a significant indirect effect on the relationship between attitude towards telemedicine and its current use (β = .14, *p* < .001). Attitudes towards telemedicine and behavioral intention to use it had an indirect effect on the relationship between perceived usefulness and current use of telemedicine (β = .30, *p* < .001). Although RMSEA indicated the trimmed version of the TRA was not as good a fit for modeling the data, the remaining fit indices suggest the trimmed version of the TAM was a good fit (Table 2) for understanding telemedicine adoption [19,20,22]. Given the only slightly diminished fit indices for the trimmed TAM, the model was retained as the best-fitting, most parsimonious model.

## Discussion

This study examined telemedicine adoption among physicians during the COVID-19 pandemic using theoretical models of behavior change. The findings suggested that the TRA was an adequate to poor fit for modeling physicians’ decisions concerning telemedicine use. While the TAM demonstrated a better fit than the TRA, a trimmed TAM was ultimately retained for parsimony with mostly good or adequate fit indices.

Results from the TRA model showed that physicians’ intention to use telemedicine was associated with their own attitude toward telemedicine and subjective norms and that subjective norms had a slightly stronger association with physicians’ behavioral intention to use telemedicine than their own attitude toward telemedicine. A shift in norms related to telemedicine use no doubt contributed to the rapid uptake of telemedicine at the beginning of the pandemic; major U.S. health organizations and agencies such as Centers for Disease Control and Prevention [23], the Department of Health and Human Services [24], and Center for Medicare and Medicaid Services [23] coordinated efforts to expand telehealth and effected rapid changes in telemedicine adoption. The official organizations’ endorsement of and support for telemedicine led to changes in subjective norms related to telemedicine use which was likely a key contributor to the accelerated uptake of telemedicine [25]. This finding highlights the critical role institutional and legislative support played in facilitating telemedicine usage and expansion.

Results from the TAM indicated that physicians’ perceived usefulness of telemedicine was positively associated with their attitude toward it and their behavioral intention to use it. While physicians’ intention to use telemedicine was influenced by both their attitudes toward it and their perceived usefulness of telemedicine in their own practice, perceived usefulness showed a stronger statistical effect. On the contrary, perceived ease of use did not significantly impact physicians’ attitude toward telemedicine or their intention to use it. These findings have been largely supported by prior literature showing that perceived usefulness is a strong predictor of healthcare providers’ technology adoption [26–29] and that perceived usefulness is generally more predictive of technology adoption than perceived ease of use [30,31]. It is perhaps not surprising that perceived usefulness was a strong motivator for physicians to adopt telemedicine during the pandemic, as telemedicine served as an alternative modality for healthcare provision during public health emergencies, which ensured safe, continued patient care access. On the other hand, the finding that perceived ease of use did not significantly influence physicians’ attitudes and intention to use telemedicine has similarly been found in prior literature [32] which may suggest that healthcare providers prioritize the usefulness and effectiveness of a system over its ease of use.

A modified version of the TAM demonstrated the best fit for the study data after perceived ease of use was omitted from the model. Physicians’ perceived usefulness of telemedicine was positively associated with their attitude toward telemedicine and their behavioral intention to use it. Their attitude toward telemedicine was also positively associated with their behavioral intention to use it, which was in turn positively associated with the actual use of telemedicine. The findings are generally in line with previous studies showing that the perceived usefulness of telemedicine strongly influenced health providers’ behavioral intention to use it [33,34]. Results from the trimmed TAM path model may inform development of targeted training programs or implementation of telemedicine practice to facilitate and expand telehealth in a post-pandemic context.

The deployment of telemedicine during the pandemic was made possible by physicians’ rapid adoption of virtual care and swift changes in policies and reimbursement around telemedicine practice. In the current post-pandemic era, despite a decline in telehealth usage, the number of telehealth encounters continue to be substantially higher than pre-pandemic levels [35], indicating a further need to integrate telemedicine into healthcare delivery and permanently modify healthcare policies and regulations to reduce care access barriers and sustain telemedicine services.

### Limitations and future directions

This study has several limitations that provide directions for future research. First, the recruitment method was prone to sampling bias [36,37]. While the majority of participants identified as women, the Association of American Medical Colleges reports that only 36.3% of physicians practicing within the U.S. in 2019 identified as women [38]. Thus, the study sample might not fully represent the characteristics of the U.S. physician population. Additionally, the study’s focus on physicians’ experiences and perceptions of telemedicine overlooked the perspective of nurse practitioners (NP) and physician assistants (PA) who also routinely provided telemedicine during the pandemic. Future studies should examine the replicability of the study findings with advanced practitioners. Second, while the survey captured physicians’ attitudes and behaviors at one point in time, the pandemic and telehealth landscape were rapidly evolving with frequent changes occurring in insurance coverage, regulations, and policies. As a result, the cross-sectional survey methodology cannot explain the causal influence of the pandemic context and related external factors on physicians’ adoption of telemedicine, and this methodology was also unable to capture physicians’ behavior change processes which were dynamic and evolving. Future research should examine barriers and facilitators of longitudinal sustainability of telemedicine. Finally, a notable limitation was the use of single-item measures to assess constructs within the TRA and TAM. While the choice of items was empirically supported, single-item measures may have inferior psychometric properties compared to multiple-item measures, and it is not possible to test the internal consistency of a single item measuring a construct. Despite the potential methodological limitations, using the original scales developed by Chau and Hu [16] likely would have resulted in an unacceptably high attrition rate due to survey burden. Future studies should consider including the full set of items to improve the psychometrics of the survey.

## Conclusions

This cross-sectional study examined physicians’ telemedicine adoption during the COVID-19 pandemic using theoretical models of behavior change. Based on the study data, the TRA and the TAM path models demonstrated an adequate fit and good fit respectively; ultimately a trimmed version of the TAM demonstrated the best fit. Results from the trimmed TAM path model may inform development of targeted training programs or implementation of telemedicine practice to facilitate and expand telehealth in the post-pandemic era.

## Supporting information

S1 File Study Survey. (DOCX)

S2 File Study Data. (SAV)
