# Supplementary material for: Examining models of telemedicine use among U.S. physicians during the COVID-19 pandemic
Source: PLoS One. 2025 Sep 8;20(9):e0331832. doi: 10.1371/journal.pone.0331832 (PMC12416656; doi:10.1371/journal.pone.0331832)
Supplement: S1 File — (DOCX) [file pone.0331832.s001.docx]

**Screener Items**

1. Are you licensed to practice as a physician in the U.S.? (yes, no)
2. Are you currently practicing (seeing patients) as a physician in the U.S.? (yes, no)
3. What is your current age (in years)?
4. For how many years have you been practicing as a physician since graduating from medical school?

**Demographic and Practice-Related Items**

1. What is your gender? (Woman, man, gender non-conforming (GNC), genderqueer, intersex, transman, transwoman, other gender [please specify])
2. What is your race/ethnicity? (American Indian/Alaska Native/Native American, Asian/Asian-American [non-Latinx/non-Hispanic], Black/African-American [non-Latinx/non-Hispanic], Latinx/Hispanic, Multiracial/Multiethnic, White/European-American [non-Latinx/non-Hispanic], Other [Please specify])
3. Would you consider your primary practice to be located in an urban, suburban, or rural setting? (Urban, suburban, rural)
4. What is your primary treatment setting? (Hospital, Veterans Affairs Hospital, Academic Medical Center, Trauma Center, Health Maintenance Organization, Correctional Facility, Geriatric Facility, Individual Practice, Group Practice, Outpatient Treatment Facility, Rehabilitation Center, Residential Treatment Facility, School/University, Other [Please Specify])
5. How many physicians (including yourself) practice within your primary treatment setting? (1, 2 – 5, 6 – 10, 11 – 20, 21 – 50, 50+)
6. What type of medicine do you practice? (Select all that apply) (Allergy and Immunology, Anesthesiology, Colon and Rectal Surgery, Dermatology, Emergency Medicine, Family Medicine, Internal Medicine, Clinical Biochemical Genetics, Clinical Genetics and Genomics, Laboratory Genetics and Genomics, Neurological Surgery, Nuclear Medicine, Obstetrics and Gynecology, Ophthalmology, Orthopedic Surgery, Otolaryngology – Head and Neck Surgery, Pathology, Pediatrics, Physical Medicine and Rehabilitation, Plastic Surgery, Aerospace Medicine, Occupational Medicine, Public Health and General Preventive Medicine, Psychiatry, Neurology, Diagnostic Radiology, Interventional Radiology and Diagnostic Radiology, Medical, Physics (Diagnostic, Nuclear, Therapeutic), Radiation Oncology, General Surgery, Vascular Surgery, Thoracic and Cardiac Surgery, Urology, Other [Please specify])

**Telemedicine Questions**

For the purpose of this survey, “telemedicine” refers the use of real-time audio (e.g., telephone) and/or video conferencing technology to provide healthcare services.

1. What percentage of your patient treatment is provided using telemedicine? (0, 10, 20, 30, 40, 50, 60, 70, 80, 90, 100)
2. Using telemedicine in patient care and management is a good idea.

Strongly Strongly

Disagree Neutral Agree

├──────┼──────┼──────┼──────┼──────┼──────┤

1. People who are important in assessing my patient care and management think that I should use telemedicine.

Strongly Strongly

Disagree Neutral Agree

├──────┼──────┼──────┼──────┼──────┼──────┤

1. Using telemedicine can improve my patient care and management.

Strongly Strongly

Disagree Neutral Agree

├──────┼──────┼──────┼──────┼──────┼──────┤

1. I find telemedicine easy to use.

Strongly Strongly

Disagree Neutral Agree

├──────┼──────┼──────┼──────┼──────┼──────┤

1. To the extent possible, I would use telemedicine in my patient care frequently.

Strongly Strongly

Disagree Neutral Agree

├──────┼──────┼──────┼──────┼──────┼──────┤

1. If I wanted to, I would have the ability to use telemedicine in my patient care and management.

Strongly Strongly

Disagree Neutral Agree

├──────┼──────┼──────┼──────┼──────┼──────┤
